# Validation of actigraphy to assess circadian organization and sleep quality in patients with advanced lung cancer

**DOI:** 10.1186/1740-3391-9-4

**Published:** 2011-05-18

**Authors:** James F Grutsch, Patricia A Wood, Jovelyn Du-Quiton, Justin L Reynolds, Christopher G Lis, Robert D Levin, Mary Ann Daehler, Digant Gupta, Dinah Faith T Quiton, William JM Hrushesky

**Affiliations:** 1 Cancer Treatment Centers of America at Midwestern Regional Medical Center, Zion, IL, USA; 2 Medical Chronobiological Laboratory, Dorn Research Institute, WJB Dorn VA Medical Center, Columbia, SC, USA; 3 School of Medicine, University of South Carolina, Columbia, SC, USA; 4 School of Public Health Cancer Prevention and Control Program, University of South Carolina, Columbia, SC, USA; 5 School of Engineering and Information Technology, University of South Carolina, Columbia, SC, USA; 6 University of Illinois School of Public Health, Chicago, IL, USA

## Abstract

**Background:**

Many cancer patients report poor sleep quality, despite having adequate time and opportunity for sleep. Satisfying sleep is dependent on a healthy circadian time structure and the circadian patterns among cancer patients are quite abnormal. Wrist actigraphy has been validated with concurrent polysomnography as a reliable tool to objectively measure many standard sleep parameters, as well as daily activity. Actigraphic and subjective sleep data are in agreement when determining activity-sleep patterns and sleep quality/quantity, each of which are severely affected in cancer patients. We investigated the relationship between actigraphic measurement of circadian organization and self-reported subjective sleep quality among patients with advanced lung cancer.

**Methods:**

This cross-sectional and case control study was conducted in 84 patients with advanced non-small cell lung cancer in a hospital setting for the patients at Midwestern Regional Medical Center (MRMC), Zion, IL, USA and home setting for the patients at WJB Dorn Veterans Affairs Medical Center (VAMC), Columbia, SC, USA. Prior to chemotherapy treatment, each patient's sleep-activity cycle was measured by actigraphy over a 4-7 day period and sleep quality was assessed using the Pittsburgh Sleep Quality Index (PSQI) questionnaire.

**Results:**

The mean age of our patients was 62 years. 65 patients were males while 19 were females. 31 patients had failed prior treatment while 52 were newly diagnosed. Actigraphy and PSQI scores showed significantly disturbed daily sleep-activity cycles and poorer sleep quality in lung cancer patients compared to healthy controls. Nearly all actigraphic parameters strongly correlated with PSQI self-reported sleep quality of inpatients and outpatients.

**Conclusions:**

The correlation of daily activity/sleep time with PSQI-documented sleep indicates that actigraphy can be used as an objective tool and/or to complement subjective assessments of sleep quality in patients with advanced lung cancer. These results suggest that improvements to circadian function may also improve sleep quality.

## Background

Living organisms use circadian (about 24-hour) oscillators and environmental cues to adjust the dynamics of their physiological/behavioral processes to critical phases of the geophysical day [1,2]. Preclinical and clinical data show that circadian organization diminishes with accelerating tumor growth and accurately predicts poor prognosis, while restoring normal circadian function improves quality of life and enhances the survival benefits of chemotherapy [3-7].

Satisfying sleep is an important sign of a robust and well-entrained endogenous circadian time structure. Poor nighttime sleep quality is associated with reduced quality of life and unremitting daytime fatigue. Each of these traits is linked to diminished cancer patient survival [8-10]. Surveys of sleep disturbances between different groups of cancer patients report prevalence rates from a low of 24% to a high of 95% [9]. These observations suggest that circadian organization has the potential to tell us a great deal about the overall health of cancer patients [7].

Wrist actigraphy is a noninvasive tool for assessing the 24-hour sleep-activity cycle by monitoring continuous non-dominant wrist movements [11]. Actigraphy has been validated with concurrent polysomnography to objectively measure many standard sleep quality and quantity parameters as well as daily activity of healthy individuals [11-15]. Care has been taken to fully specify the instrumentation type, sampling mode and analysis tools in order to allow inclusion of this study in the growing database of cancer studies using actigraphy [16].

This report investigates the hypothesis that advanced lung cancer patients' circadian activity rhythm correlates with patient's self report of nighttime sleep quality. This report also assesses whether chronic obstructive pulmonary disease (COPD) status and severity confounds the relationship between self-report of sleep quality and their measured circadian function among advanced lung cancer patients.

The primary goal of the study is to determine whether and how the circadian organization of cancer patients is affected by the cancer-bearing state. The secondary goal is to determine whether and how objective measurement of activity and sleep using actigraphy can quantify cancer-associated circadian disruption. The tertiary goal is to determine the relationship between these objective measurements of circadian organization and subjectively reported nighttime sleep and daytime fatigue. Finally, we assess, whether and how hospitalization and chronic obstructive lung disease mask these circadian relationships.

## Methods

### Protocol Summary

The study was conducted concurrently at Cancer Treatment Centers of America (CTCA) at Midwestern Regional Medical Center (MRMC), Zion, Illinois, USA and the WJB Dorn Veterans Medical Center (VAMC), Columbia, South Carolina, USA, from June 2002 to April 2006. Forty-two eligible patients who were about to undergo chemotherapy for advanced lung cancer were enrolled at each site. All patients were asked to complete the Pittsburg Sleep Quality Index (PSQI) questionnaire prior to their first chemotherapy treatment. For the MRMC patients, actigraphy was performed at the inpatient setting before and during their first chemotherapy cycle, while for the VAMC patients, actigraphy data were obtained in the outpatient/home setting prior to the initiation of chemotherapy. Henceforth, we refer to MRMC patients as *inpatients *while VAMC patients as *outpatients*. Actigraphic data of healthy controls were obtained from the Ambulatory Monitoring, Inc (AMI) database. Presence and severity of COPD was obtained through clinical review of the current medical records of the patients in VAMC. This information was not available for MRMC inpatients.

### Patients

Patients, between the ages of 18 and 94 were studied. Each had a pathologically confirmed diagnosis of advanced stage (IIB, IIIA, IIIB, IV) or recurrent non-small cell lung cancer (NSCLC), with either bidimensionally measurable or evaluable unresectable disease, including histologically positive ascites and histologically positive pleural effusion, and an Eastern Cooperative Oncology Group (ECOG) performance status of 0, 1, or 2. ECOG scores stratify patient's performance status on a scale of 0 (denoting perfect health) to 5 (dead). In this investigation, patients were restricted to scores of 0, 1 (fully active but symptomatic), and 2 (capable of self-care and able to carry out work of a light or sedentary nature). Untreated patients and patients who had failed one prior chemotherapy treatment regimen were eligible. Ineligible patients included those with medical conditions that precluded administration of chemotherapeutic agents, such as inadequate renal function with serum creatinine > 221 mmol × 10^-1^, inadequate hepatic function with bilirubin > 34.2 mmol × 10^-1^, uncontrolled congestive heart failure; uncontrolled hypertension, arrhythmia, or angina; carcinomatous meningitis; or uncontrolled infection. Patients with a history of brain metastases, or another uncontrolled primary cancer were ineligible. All patients signed an Informed Consent indicating that they were aware of the investigational nature of the study. The Institutional Review Boards at MRMC and VAMC approved the study. This current report is based on data obtained at initial enrollment.

### Actigraphy Measurements of Sleep/Activity Cycles

A watch-like wrist actigraph, worn on the non-dominant wrist, was used to record a patient's level and pattern of gross motor activity (Mini Motionlogger Basic model, Ambulatory Monitoring, Inc, AMI). Internal motion sensors capture patient movement data, measured as the number of accelerations per minute (Zero Crossing Mode). Sleep is reflected by spans without accelerometer movements as validated by AMI using formal sleep lab studies. These movement data are transferred to a computer for analysis to produce a report containing parameters of sleep and wake periods, their timing, duration and other characteristic details. For each patient, the following parameters were used to describe the activity phase of the daily circadian cycle: mean daily activity (activity mean), mean duration of activity during conventional wake periods (wake minutes), mean duration of sleep during conventional wake periods (sleep minutes), proportion of conventional wake periods spent sleeping (% sleep), number of sleep episodes during conventional wake periods (sleep episodes), frequency of long naps (long sleep episodes > = 5 minutes). During the presumed sleep phase of the circadian cycle, the following parameters were evaluated: mean duration of wakefulness (wake minutes), number of sleep interruptions (wake episodes), frequency of long sleep interruptions (long wake episodes > = 5 minutes), proportion of sleep span spent actually sleeping (% sleep), sleep latency, sleep efficiency, frequency of long sleep episodes (long sleep episodes).

### Site Differences in Actigraphy

Each patient's baseline sleep/activity cycle was measured prior to or during the first cycle of therapy, to achieve a minimum of 48 hours of high quality continuous activity data. The timing and conditions of actigraphy measurement were necessarily different at each of the two sites. Because MRMC is a tertiary cancer center, actigraphy data were recorded in the in-patient setting prior to and often during the administration of the first cycle of chemotherapy. Actigraphy was recorded in the patient's home for 4-7 days in VAMC patients. The difference in activity between in- and out-patients is substantial and confounding. Consequently, all analyses of actigraphic wake/sleep parameters are stratified by site. There were no site differences in prior treatment, cancer stage, and ECOG performance status.

### Patient Therapy

All patients received identical chemotherapy consisting of Cisplatin 25 mg/m^2 ^and Etoposide 100 mg/m^2 ^each on days 1, 2, and 3. This regimen was repeated every 28 days.

### Determination of Presence and Severity of COPD

COPD, which is present in the majority of lung cancer patients, is a potential confounding variable for this investigation of sleep and circadian time structure. All outpatients, but no inpatients, were assessed clinically and with pulmonary function tests for the presence of COPD. Its severity was graded according to the Spirometric Classification of COPD severity, by reference to percent of predicted forced expiratory volume in one second (FEV_1_). Thirty to 50% percent of predicted FEV_1 _is considered severe; moderate is 50% to 89% percent; and mild COPD is greater than 80% of predicted FEV_1._No such data are available for MRMC patients.

### PSQI

Patient's sleep quality was assessed through the PSQI, which is a questionnaire that assesses sleep quality and quantity over a one-month span. The PSQI contains 19 items that comprise an overall sleep score. It produces separate scores in seven component domains: subjective sleep quality, sleep latency, sleep duration, habitual sleep efficiency, sleep disturbances, use of sleep medication, and daytime dysfunction. The seven component scores are totaled to produce a *Global Sleep Quality Score *for each patient. The questionnaire requires the patient to describe patterns of sleep such as typical bedtime and wake time, length of time taken to fall asleep, and actual sleep time. The patient then answers a series of questions relating to sleep habits and quality. Component scores are based on a four-point Likert scale that ranges from Very Good (0) to Very Bad (3). The component scores are combined to produce the *Global Sleep Quality Score *ranging from 0 to 27. Those having a score greater than 5 are considered poor sleepers, but among cancer patients those with a score greater than eight have been considered poor sleepers [17].

### Statistical Analysis

Descriptive statistics were computed for numeric demographic factors and actigraphy endpoints to describe the average and variability of the population. Frequency and percentages were computed for qualitative factors such as sex. Either parametric or non-parametric analysis of variance, whichever was appropriate, was used to determine differences among factor levels (SAS v 9.1, Cary, NC). For four to seven days, an actigraphy watch recorded the number of accelerations per minute. This data was translated into sleep/activity parameters through the Act Millenium and Action W2 software (Ambulatory Monitoring, Inc). Rhythmometric analysis (using Chronolab v2) was done on these sleep/activity patterns in order to assess disruption and consolidation of sleep in lung cancer patients. Rhythmometric analysis fits a cosine curve to the circadian activity providing three standard parameters: mesor (the average activity over the 24-hr period), amplitude (1/2 peak to nadir difference) and acrophase (the time of peak activity). In addition to these parameters, we also computed the circadian quotient (amplitude/mesor) to characterize the strength of the circadian rhythm and the rhythm quotient [A_24 HR_/(A_4_+A_8_+A_12_)]. In our patients, higher amplitudes are often associated with more robust rhythms; for example, people who move vigorously during the day and sleep soundly during each night would have higher amplitudes. The circadian quotient provides normalized values that would allow comparison between individuals [18,19]. Activity patterns of normal people usually have 1 or 2 major circadian components and best rhythm fit are 24 hours or 12 hours. The rhythm quotient provides a basis for the quality of circadian rhythms and how well activity and sleep are each consolidated within the day. Higher rhythm quotient indicates a more pronounced circadian rhythm and lower values indicate fractured sleep-activity patterns. Further, circadian rhythms were assessed through spectral density analysis where 24-hr autocorrelations (r*_24_*) were computed. Autocorrelations theoretically can range from -1 to +1. If a circadian variation is present, autocorrelations will increase near the 24-hour period and a more pronounced circadian rhythm will result in a higher autocorrelation at 24-hour. Aside from these parameters, day-night balance of activity as well as sleep was also calculated. Day-Night Activity balance is the ratio of amount of activity during the day versus activity during the night, similarly, ratios of sleep during the night over sleep during the day is called the Night-Day Sleep balance.

### Cosinor Analysis

To uncover underlying daily rhythms and describe the shape and relationships of these recurring patterns across time in the data sets, each time series was analyzed for about 24 hours [20], with use of the Chronolab statistical package [21]. This method of time series analysis tests for the presence of a cosine-shaped pattern of an a priori defined period length in each data set. If significant, it confirms the presence of a recurring cycle or rhythm in the data, as opposed to random variation or a trend occurring across the entire observation span. Cosinor analysis is analogous to the linear regression testing by ''least squares'' of a best-fitting straight line to a data set when searching for a linear increasing or decreasing trend and subsequently determining the probability that the slope of the best-fitting line is different from zero. Using the same technique, the cosinor method fits a best-fitting cosine function instead of a straight line. The probability that the amplitude of the cosine function best fitting these data is greater than zero is calculated based upon the reduction in variance about the fitted cosine compared to the total variance about the arithmetic mean (flat line). If the zero-amplitude hypothesis can be rejected with 95% certainty, statistical significance of a modulation that approximates the length (period) of the cosine is accepted at p < = 0.05. Rhythm parameters of ''mesor,'' ''acrophase,'' and ''amplitude'' can then be derived from the cosine model used. The ''mesor'' is the mean of the rhythm and represents the middle value of the fitted cosine. The series mesor and mean are identical if the data are equidistant across the sampling span, but they are not identical if sampling is irregular or the time span is not an integral number of the longest period being fitted, or both. The ''acrophase'' is the time from a phase reference (08) to the peak of the cosine function that best describes the data. In our analyses, the fitted period, 24 hours, is referenced to local midnight as 0 degrees to 360 degrees the next local midnight. The ''amplitude'' is the height of the best-fitting cosine function from the mesor to the acrophase and is one-half of the full variation from trough to peak of the co-sine, which indicates a predictable range of change.

## Results

### Patient Actigraphy, PSQI Data and Site Characteristics

There were systematic institutional differences in demographic and clinical status of participants between the two sites (Table 1A and 1B). All forty-two patients from VAMC were males while only 23 of 42 patients from MRMC were males. VAMC patients were older; with a mean age of 66 compared to MRMC patients mean age of 57 years. Fifty percent and 26% from MRMC and VAMC, respectively, had failed previous cancer treatment. Twelve actigraphs were worn for less than 48 hours and/or had missing observations, due to instrument malfunction. Out of the 72 patients with complete actigraph recordings, four patients failed to respond to the PSQI questionnaire, so we have complete actigraphy and questionnaire data for 68 (35 inpatients, 33 outpatients) of the 84 enrolled patients.

**Table 1:** Distribution of demographic/clinical traits by site and summary of PSQI scores

1A				
	All Patients	Inpatients	Outpatients	Site Effect
**Demographic/Clinical**	**(n = 84)**	**(n = 42)**	**(n = 42)**	**(χ^2^, p)^a^**

Age in years (Mean; Range)	62 (40-94)	57(40-78)	66(47-94)	4.0, <0.01
Sex (M:F)^b^	65:19	23:19	42:00:00	24.6, <0.01
Cancer Stage (IIB:IIIA&B: IV)^b^	1:18:65	0:10:32	1:08:33	NS
Prior Therapy (Yes:No)^b^	31:52	21:20	10:32	NS
WHO ECOG (0:1:2)^b^	30:42:11	17:18:07	13:24:04	NS
COPD (No: Mild: Mod: Severe)	ND	ND	14:7:13:8	ND

1B				

	**All Patients**	**Inpatients**	**Outpatients**	**Site Effect**

**PSQI Sleep Factor**	**(n = 64)**	**(n = 37)**	**(n = 35)**	**(t, p)**^**a**^

Sleep Quality	1.40 ± 0.11	1.23 ± 0.14	1.56 ± 0.16	NS

Sleep Latency	1.48 ± 0.12	1.46 ± 0.16	1.50 ± 0.18	NS

Sleep Duration	1.63 ± 0.14	1.62 ± 0.20	1.63 ± 0.20	NS

Sleep Efficiency	1.65 ± 0.16	1.57 ± 0.23	1.74 ± 0.21	NS

Sleep Disturbance	2.11 ± 0.12	1.80 ± 0.17	2.30 ± 0.15	5.6, 0.02

Sleep Medication	0.78 ± 0.12	0.81 ± 0.17	0.75 ± 0.17	NS

Daytime Dysfunction	1.34 ± 0.13	1.16 ± 0.16	1.52 ± 0.20	NS

Global Sleep Quality Score	11.19 ± 0.66	10.86 ± 0.93	11.54 ± 0.94	NS

^a ^Based on t-test (t, p-value). ^b ^Values are numbers of patients. ^c ^Owen et al (1999) 26, 1649-51; NS = not significant; ND = no data available

^a ^Based on t-test (t, p-value)

### Patient Provided Sleep Outcomes by PSQI

**Figure 1:**
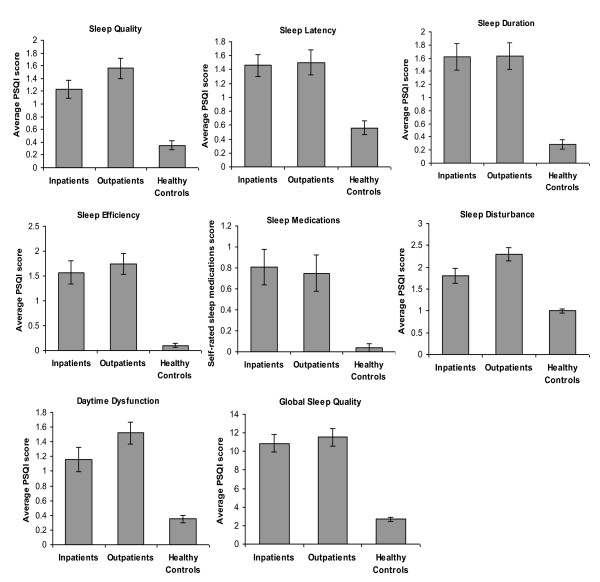
**PSQI-measured sleep quality differences between inpatients, outpatients and healthy controls**. Lung cancer patients demonstrate poorer sleep quality, quantity and more daytime dysfunction when compared to healthy subjects.

Lung cancer patients' mean Global PSQI score was 11.19 ± 0.66, which exceeds the threshold score of 8 for poor quality sleep (Table 1) [17]. PSQI scores of lung cancer patients demonstrate poorer sleep quality, sleep latency, sleep duration, sleep efficiency, and more daytime dysfunction and sleep disturbance when compared to healthy controls (Figure 1).

There was no significant difference in sleep quality by site; 83.88% of MRMC patients had a Global PSQI score of 5 or more and 64.86% had score of at least 8, while 85.71% of VAMC patients had Global PSQI score of at least 5 and 82.86% had score of at least 8. Only sleep disturbance differed by site, where outpatient scores were statistically significantly worse than inpatients (χ^2 ^= 5.6, p = 0.02; Table 1).

**Figure 2:**
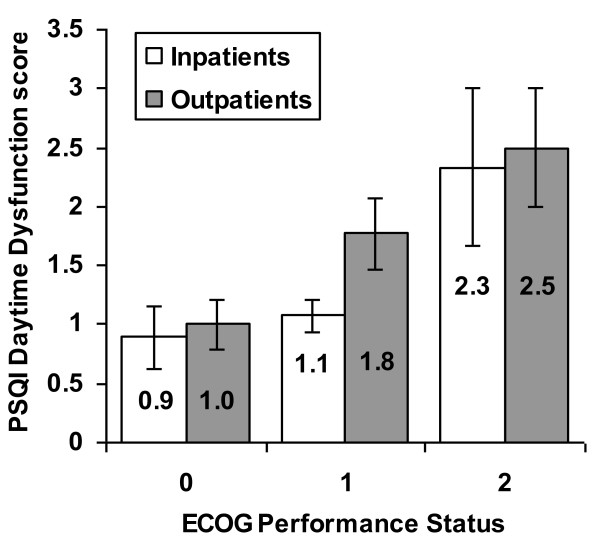
**Among both inpatients and outpatients, the relationship between ECOG performance status and PSQI domain score in daytime dysfunction worsened with worsening performance status score**.

There were statistically significant associations between ECOG performance status and sleep disturbance (e.g., nightmares, breathing difficulty, etc; χ^2 ^= 4.1, p = 0.04, Figure 2) and greater daytime dysfunction (e.g., staying awake while working, driving etc; χ^2 ^= 8.3, p = 0.02; data not shown).

### Concomitant Relevant Illness

COPD and lung cancer share a common etiology and produce similar symptoms. Consequently, they each potentially affect the patients' sleep quality. In outpatients, 67% suffered documented COPD, 20% (8 of 42) had severe, 31% (13 of 42) had moderate and 16% (7 of 42) had mild COPD (Table 1). Two of the 27 measured PSQI components had a statistically significant association with COPD severity; global PSQI score (two-sided Fisher's Exact test, p = 0.0238; data not shown) and habitual sleep efficiency (two-sided Fisher's Exact test, p = 0.0022; data not shown). The presence and severity of COPD did not affect any of the relationships of actigraphic circadian organization and sleep quality.

### Actigraphy Lung Cancer Patient Data Compared To Normal Controls

Actigraphic parameters of all cancer patients during the Wake Period and the Sleep Period, from both sites, were considered grossly abnormal when compared to healthy individuals (Action-W v.2 database, Ambulatory Monitoring, Inc.). This control database is comprised of 3-day actigraphy measurements of 35 adults, aged 20-50 years having no known disease.

**Figure 3:**
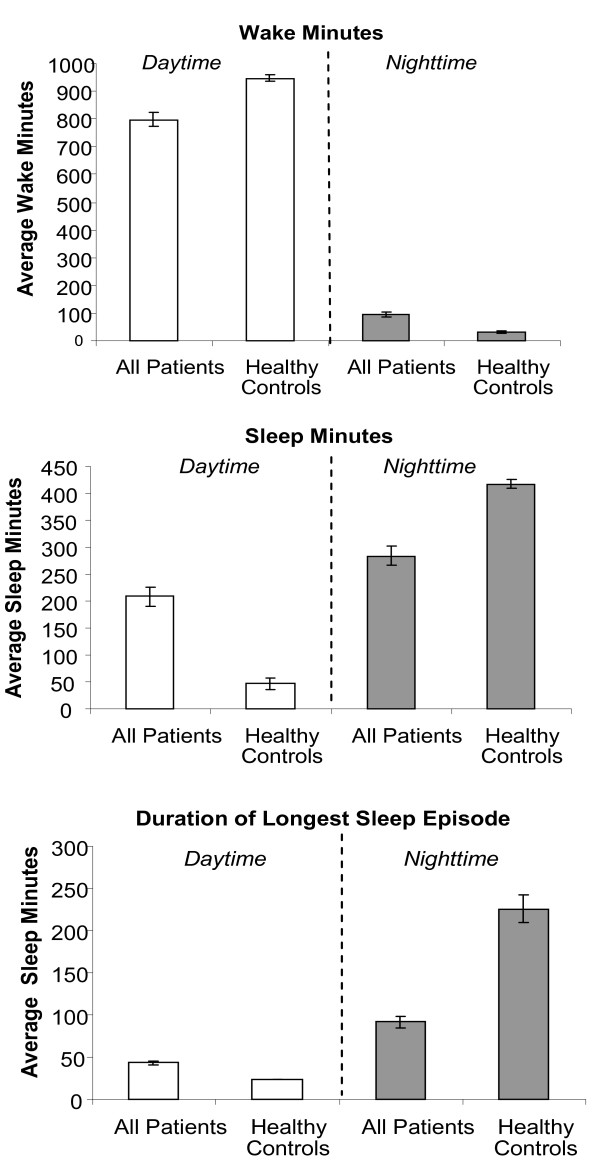
**Objective actigraphic parameters that illustrate daytime dysfunction among cancer patients when compared to healthy controls**.

During the Wake Period of putative activity, cancer patients were 20 to 50% less active than the controls (Table 2; Figure 3). The patients were inactive or napping at least three times longer than the controls (% sleep: 20.9% versus 4.7%) and these episodes of inactivity or napping were longer than those occurring in healthy individuals. During the nightly sleep span, lung cancer patients had more and longer waking episodes than controls. The duration of nighttime sleep for the patients was diminished by 35% compared to controls and the duration of the longest sleep episode was approximately 40% of controls. There were no gender differences in any actigraphic parameter among inpatients, where females were studied.

**Table 2:** Actigraphic activity-sleep characteristics during the wake period and sleep period of non-small cell lung cancer patients compared to population-based controls

	Wake Period		Sleep Period		By Site	
**Actigraphic Parameters**	**All patients**	**Population controls**	**All patients**	**Population controls**	**Inpatients**	**Outpatients**

Cases	68	35	68	35	35	33

Mean activity(accel/min)	126.9 ± 4.9*	182.6 ± 25	ND	ND	111.7 ± 7.1	143.0 ± 5.6

Wake minutes	797.5 ± 26*	947.1 ± 10.9	95.0 ± 8.8*	31.1 ± 3.6	714.2 ± 36	885.8 ± 31

Sleep minutes	208.8 ± 18*	47.1 ± 10.9	284.0 ± 18.3*	417.8 ± 9.4	241.3 ± 25	174.4 ± 24

% Sleep	20.9 ± 1.8*	4.7 ± 0.7	72.5 ± 2.0*	93.0 ± 0.8	25.8 ± 2.8	15.6 ± 1.9

Duration of longest sleep (min)	43.0 ± 2.8*	23.6 ± 0.6	91.7 ± 7.4*	225.6 ± 17	45.4 ± 4.0	40.5 ± 3.9

Sleep Latency	NA	NA	20.8 ± 2.5*	12.1 ± 6.9	NA	NA

Sleep Efficiency	NA	NA	79.8 ± 1.7*	95.9 ± 0.7	NA	NA

*p < 0.05 compared to controls

Actigraphic circadian organization differed by site (Table 2). Outpatients were, on average, much more active than inpatients during the day and they consolidated activity much better than the inpatients. During the sleep phase, actigraphy at both sites were indistinguishable. These prominent site differences in actigraphy collection protocols required that the data be analyzed by site.

### Correlation between Actigraphy and PSQI

#### Usual Wake Period

**Figure 4:**
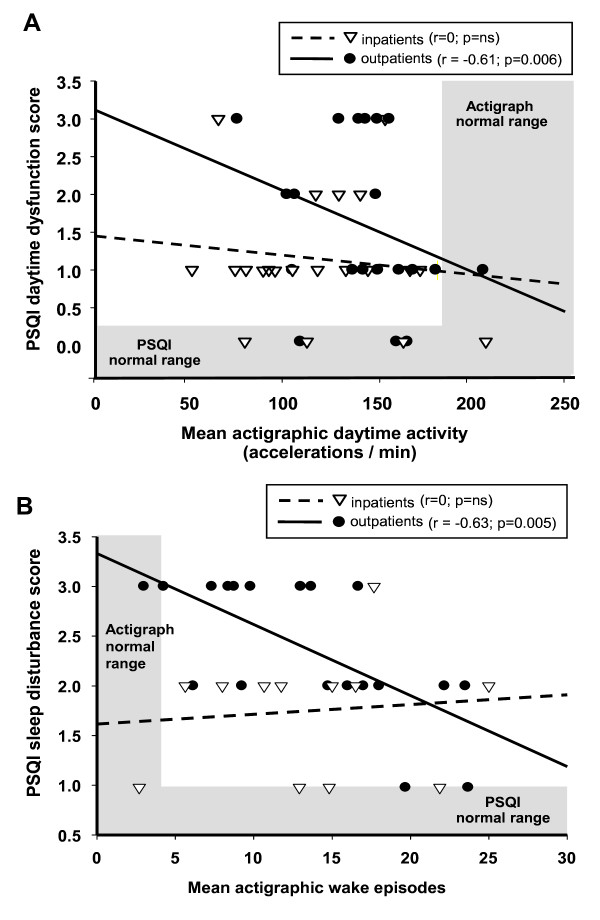
**Relationship of Subjective (PSQI) and Objective (Actigraphy) assessments of activity (A) and wakefulness during sleep (B)**. Correlations between the two assessments are the most robust among outpatients, while actigraphic parameters were potentially masked in inpatients.

Nearly all actigraphy parameters measured in outpatients during the usual Wake Period correlated with PSQI self-reported measures of sleep quality, but only a few parameters correlated among inpatients. Among outpatients, there were statistically significant correlations between patients' levels of daytime activity and lower use of sleep medication as self-reported in the PSQI (r = -0.58, p < 0.01; Table 3), lower PSQI reported day time dysfunction (r = -0.61, p < 0.01) and better overall PSQI sleep quality (r = -0.48, p = 0.01). Among inpatients, more daytime inactivity (sleep minutes) was associated with higher self-reported use of sleep medications (r = 0.39, p = 0.05), more daytime dysfunction (r = 0.54, p = 0.02) and lower PSQI global sleep quality (r = 0.41, p = 0.04) (Table 3). Two PSQI measures are plotted against two corresponding actigraphy parameters to demonstrate the correlation (Figure 4).

**Table 3:** Correlation of PSQI components and Actigraphy during the Usual Wake Period by Site^a^

Actigraphy Parameters (Wake Period)	PSQI Sleep Medicine Use	PSQI Daytime Dysfunction	Global PSQI Score
***Inpatients ***(n = 35)			
Activity Mean	ns	ns	ns
Sleep Minutes	0.39(0.05)	ns	ns
% Sleep	-0.37(0.064)	ns	ns
Wake Episodes	ns	ns	ns
Mean Wake Episode	ns	ns	ns
Long Wake Episode	ns	-0.46(0.03)	ns
Sleep Episodes	ns	ns	ns
Mean Sleep Episode	-0.41(0.035)	ns	ns
Long Sleep Episode	ns	ns	ns
Longest Sleep Episode	-0.41(0.04)	ns	ns

***Outpatients ***(n = 33)			
Activity Mean	-0.58(0.003)	-0.61(0.006)	-0.48(0.014)
Sleep Minutes	ns	0.54(0.017)	0.41(0.036)
% Sleep	ns	0.45(0.053)	0.37(0.06)
Wake Episodes	0.40(0.047)	ns	ns
Mean Wake Episode	-0.52(0.008)	ns	-0.43(0.027)
Long Wake Episode	0.34(0.096)	ns	ns
Sleep Episodes	0.40(0.047)	ns	0.35(0.078)
Mean Sleep Episode	ns	0.62(0.004)	ns
Long Sleep Episode	ns	0.46(0.047)	0.43(0.029)
Longest Sleep Episode	ns	0.61(0.005)	0.45(0.02)

^a ^Correlations are shown only for p-values < 0.05; ns = not significant; p-values are in ( ).

#### Conventional Sleep Period

There were statistically significant correlations between actigraphy parameters measuring sleep and the PSQI parameters of sleep duration, sleep efficiency, sleep disturbance, sleep medication, daytime dysfunction and global PSQI sleep quality (Table 4). Among outpatients, the number of wake episodes during the night was associated with more sleep disturbance (r = 0.63, p < 0.01) and daytime dysfunction (r = 0.55, p = 0.02), but it was associated with more sleep medication among inpatients (r = 0.34, p = 0.09; Table 4). Wake after sleep onset is significantly associated with poorer global sleep quality studied in these patients homes (r = -0.46, p = 0.02). The duration of sleep latency is correlated with the use of sleep medication in both inpatients (r = 0.62, p < 0.01) and outpatients (r = -0.38, p = 0.06). Furthermore, for outpatients, there were significant correlations between actigraphically-measured nighttime sleep episodes and the PSQI parameters of sleep disturbance (r = -0.63, p < 0.01), daytime dysfunction (r = -0.57, p = 0.01) and global sleep quality (r = -0.49, p = 0.01). These associations were apparently masked by hospitalization.

**Table 4:** Correlation of PSQI components and Actigraphy during the Usual Sleep Period

Actigraphy Parameters (Sleep Period)	PSQI Sleep Disturbance	PSQI Sleep Medicine Use	PSQI Daytime Dysfunction	Global PSQI Score
***Inpatients ***(n = 35)				
Wake Minutes	ns	0.44 (0.025)	ns	ns
Wake Episodes	ns	0.34 (0.09)	ns	ns
Mean Wake Episode	ns	0.40 (0.043)	ns	ns
Long Wake Episode	ns	0.47 (0.014)	ns	ns
Longest Wake Episode	ns	0.41 (0.038)	ns	ns
Wake After Sleep Onset	ns	0.35 (0.077)	ns	ns
Sleep Latency	ns	0.62 (< 0.001)	ns	ns
Sleep Efficiency	ns	ns	ns	ns
Sleep Episodes	ns	041 (0.038)	ns	ns
Long Sleep Episode	ns	ns	ns	ns

***Outpatients ***(n = 33)				
Wake Minutes	ns	ns	ns	ns
Wake Episodes	0.63 (0.005)	ns	0.55 (0.015)	0.49 (0.01)
Mean Wake Episode	ns	ns	ns	ns
Long Wake Episode	ns	-0.43 (0.031)	ns	ns
Longest Wake Episode	ns	ns	ns	ns
Wake After Sleep Onset	ns	ns	ns	-0.46 (0.018)
Sleep Latency	ns	-0.38 (0.058)	ns	ns
Sleep Efficiency	ns	ns	ns	ns
Sleep Episodes	-0.63 (0.005)	ns	-0.57 (0.011)	-0.49 (0.011)
Long Sleep Episode	-0.53 (0.023)	ns	-0.47 (0.043)	-0.41 (0.035)

^a ^Correlations are shown only for p-values < 0.05; ns = not significant; p-values are in ( ).

#### Actigraphic Circadian Parameters

**Figure 5:**
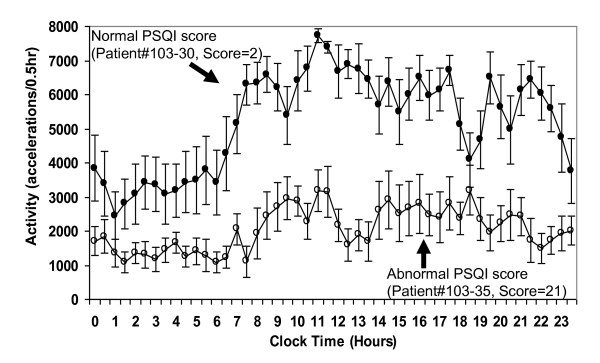
**Actigraphy pattern of two patients who had normal and abnormal PSQI Global Sleep Scores**. The 24 hr pattern of activity of a lung cancer patient who had an overall PSQI Global Sleep Score of 2 (normal, upper curve) is more rhythmic than the flattened daily activity pattern of a patient who scored 21 (abnormal, lower curve) on the overall PSQI Global Score.

Activity and sleep, considered together, create daily sleep-activity rhythms. In outpatients, higher daily mean activity is associated with lower sleep medication use (r = -0.45, p = 0.02; Table 5) and a higher circadian amplitude of activity is associated with less daytime dysfunction (r = -0.45, p = 0.05). Moreover, outpatients who exhibit higher 24-hour rhythm quotients suffer less daytime dysfunction (r = -0.58, p < 0.01), while these associations are not evident among hospitalized patients (Table 5). Patients who sleep less during the day and consolidate sleep well during the night, as measured by Day-Night Sleep Balance, sleep longer, regardless of study site (inpatients: r = 0.43, p = 0.016; outpatients: r = 0.43, p < 0.03). Higher levels of night-day sleep balance are likewise associated with less nighttime sleep disturbance (r = -0.44, p = 0.067), less day time dysfunction (r = -0.43, p = 0.065) and better global PSQI sleep (r = -0.36, p = 0.071) in outpatients, but not in inpatients (Table 5). Table 6 illustrates all relationships that occur when data for both sites are combined. These overall relationships are the most robust as they occur across both sites. To illustrate the relationship between PSQI and actigraphy, we contrasted the circadian rhythm of activity (accelerations/0.5 hr) in a patient with a normal Global PSQI score and a patient with a typically poor Global PSQI score (Figure 5). We also demonstrate the differences in 3 actigraphic sleep/wake parameters between the study patients and healthy controls.

**Table 5:** Correlations of PSQI Components and Actigraphy Parameters of Circadian Organization for Inpatients and Outpatientsa

Actigraphy Parameters (Circadian)	PSQI Sleep Duration	PSQI Sleep Efficiency	PSQI Sleep Disturbance	PSQI Sleep Medicine	PSQI Daytime Dysfunction	PSQI Overall PSQI
**Inpatients**(n = 35)						
24 HR rhythm Mean	ns	ns	ns	ns	ns	ns
24 HR rhythm Amplitude	ns	ns	ns	ns	ns	ns
Peak Activity	ns	ns	ns	ns	ns	ns
Circadian Quotient	ns	ns	ns	ns	ns	ns
Rhythm Quotient	ns	ns	ns	ns	ns	ns
Day-Night Activity Balance	ns	ns	-0.61 (0.037)	ns	ns	ns
Day-Night Wake Balance	ns	0.4(0.03)	ns	ns	ns	ns
Day-Night Sleep Balance	-0.43 (0.016)	ns	ns	0.46 (0.018)	ns	ns
Night Day Long Sleep Balance	0.37 (0.039)	ns	ns	ns	ns	ns
Night Day Longest Sleep Balance	-0.38 (0.03)	ns	ns	ns	ns	ns
Night-Day Sleep Balance	ns	ns	ns	ns	ns	ns

***Outpatients ***(n = 33)						
24 HR rhythm Mean	ns	ns	ns	-0.45 (0.02)	ns	ns
24 HR rhythm Amplitude	ns	ns	ns		-0.45 (0.048)	ns
Peak Activity	ns	ns	ns	-0.45 (0.048)	ns	ns
Circadian Quotient	ns	ns	ns	ns	-0.42 (0.065)	ns
Rhythm Quotient	ns	ns	ns	ns	-0.58 (0.007)	ns
Day-Night Activity Balance	ns	ns	-0.61 (0.037)	ns	ns	ns
Day-Night Wake Balance	ns	0.36 (0.08)	ns	ns	ns	ns
Day-Night Sleep Balance	0.43 (0.027)	ns	ns	ns	-0.62 (0.004)	-0.49 (0.01)
Night Day Long Sleep Balance	0.37 (0.063)	ns	-0.42 (0.08)	ns	-0.64 (0.003)	-0.52 (0.006)
Night Day Longest Sleep Balance	0.43 (0.028)	ns	ns	ns	-0.51 (0.027)	-0.4 (0.044)
Night-Day Sleep Balance	ns	ns	-0.44 (0.067)	-0.37 (0.07)	-0.43 (0.065)	-0.36 (0.071)

^a ^Correlations are shown only for p-values < 0.05; ns = not significant; p-values are in ( ).

**Table 6:** Correlation of Circadian Actigraphy Parameters and PSQI of NSCLC Patients

Actigraphy Parameters	Pittsburgh Sleep Quality Index									
	
	Sleep Duration		Sleep Disturbance		Sleep Medicine Use		PSQI Daytime Dysfunction		Global Score	
			
	Inpatients (n = 35)	Outpatients (n = 33)	Inpatients (n = 35)	Outpatients (n = 33)	Inpatients (n = 35)	Outpatients (n = 33)	Inpatients (n = 35)	Outpatients (n = 33)	Inpatients (n = 35)	Outpatients (n = 33)
***Wake Span***										

Activity Mean					0.07	-0.58(0.003)	-0.25	-0.61(0.006)	-0.11	-0.48(0.014)

Sleep Minutes					0.39(0.05)	-0.36	0.09	0.54(0.017)	0.26	0.41(0.036)

Mean Wake Episode					0.05	-0.52(0.008)	-0.29	0.23	-0.11	-0.43(0.027)

Sleep Episodes					-0.12	0.40(0.047)	0.18	0.28	0.08	0.35(0.078)

Mean Sleep Episode					-0.41(0.035)	-0.08	-0.31	0.62(0.004)	0.18	0.26

***Sleep Span***										

Wake Episodes			0.37	0.63 (0.005)	0.34 (0.09)	-0.23	0.25	0.55 (0.015)	-0.32	0.49 (0.01)

Sleep Latency			0.13	0.29	0.62 (< 0.001)	-0.38 (0.058)	0.11	-0.21	-0.04	0.26

Sleep Efficiency			-0.34	0.02	-0.35	0.31	0.02	0.01	0.12	0.22

Sleep Episodes			0.37	-0.63 (0.005)	041 (0.038)	-0.24	0.25	-0.57 (0.011)	-0.28	-0.49 (0.011)

***Circadian Parameters***										

24 HR rhythm Mean						-0.45 (0.02)				

Circadian Quotient								-0.42 (0.065)		

Rhythm Quotient								-0.58 (0.007)		

Day-Night Activity Balance				-0.61 (0.037)						

Day-Night Sleep Balance		0.43 (0.027)			0.46 (0.018)			-0.62 (0.004)		-0.49 (0.01)

Night Day Long Sleep Balance		0.37 (0.063)		-0.42 (0.08)				-0.64 (0.003)		-0.52 (0.006)

^a ^Correlations are shown only for p-values < 0.05; NSCLC = non-small cell lung cancer

#### Correlation between COPD and Actigraphy

No statistically significant association was found between any actigraphic parameter of activity or sleep and COPD presence or severity in this patient population in which this potential covariate was recorded. Post traumatic stress disorder (PTSD) effects could not be discovered as only two of the eighty four patients were diagnosed with this syndrome.

## Discussion

Actigraphy measurements confirm patient self-report of abnormal sleep quality and correlate with one another. Our patients' mean nocturnal sleep span is 4.7 hours compared to the adult normal sleep span of seven to nine hours [22]. Healthy adults take less than 20 minutes to fall asleep after going to bed, but our patients took more than twice as long [23]. Normally adults awaken two to six times per night and remain awake for a total of less than 40 minutes [24,25], but our patients' mean awake time during the nighttime was 95 minutes. Daytime inactivity in our control population was 46.5 minutes, while our patients' daytime napping time was 3.5 hours/day. Finally, the patients' daily activity rhythm for both sites was severely damped in comparison to the population-based control group.

All patients' PSQI scores reveal poor quality sleep. There were strong correlations between the severity of daily activity-sleep time structure abnormalities and self-reported PSQI scores. These correlations indicate that the actigraphic measure of sleep and activity can accurately and quantitatively confirm the patient self-report of sleep quality.

In addition to a dysfunctional circadian activity rhythm, many of the patients have COPD, which can contribute to insomnia and sleep maintenance problems. Although two of the seven components of the PSQI showed a statistically significant association with increasing COPD severity, there was no correlation between COPD and any actigraphy parameter. COPD, therefore, influences patients' sleep quality independently of the host's circadian function.

Our investigation has several significant limitations. Our clinics could not provide gender and aged-matched controls, but the population-based control illustrates the extent of our patients' abnormal circadian function. A second limitation is that actigraphy was measured under different circumstances at each study site. One site used actigraphy for inpatients 1-2 days before and while undergoing cancer therapy, while the other site recorded actigraphy in the patients' homes, before the initiation of any treatment. This limitation has, however, produced a valuable insight in hospitalized lung cancer patients--the variation in all day/night patterns and rhythms are so suppressed by hospitalization that most relationships between the patients' self-report of daytime activity and sleep quality and actigraphy-measured activity and sleep function are masked in this setting. The hospital routine obviously changes the daily activity pattern obscuring some of these circadian rhythms.

## Conclusions

Actigraphy as a quantitative measure of circadian disruption is of growing utility since circadian disruption has been shown to increase risk for breast, colon, prostate and endometrial cancer [26-29]. Our findings suggest that outpatient actigraphy is an effective tool to quantitatively assess whether a patients' disrupted sleep is due to a dysfunctional circadian organization of activity and rest. These results suggest that treatments designed to improve circadian function may also improve sleep quality, daytime function, diminish daytime fatigue, and enhance cancer patients' quality of life. The next step is to try to improve circadian organization of cancer patients: behaviorally with morning exercise; pharmacologically with evening melatonin or photodynamically with morning light therapy among other circadian tuning strategies.

## Competing interests

The authors declare that they have no competing interests.

## Authors' contributions

JFG, PAW, WJMH, CGL, RDL, and MAD participated in concept and design of this investigation. PAW, JLR, MAD, and DTQ recruited patients and data acquisition and interpretation. PAW, JDQ, JFG, WJMH, DG participated in concept, statistical analysis, data interpretation and writing. All authors read and approved the final manuscript.
